# Phenotypic Space and Variation of Floral Scent Profiles during Late Flower Development in *Antirrhinum*

**DOI:** 10.3389/fpls.2016.01903

**Published:** 2016-12-21

**Authors:** Julia Weiss, Joëlle K. Mühlemann, Victoria Ruiz-Hernández, Natalia Dudareva, Marcos Egea-Cortines

**Affiliations:** ^1^Department of Genetics, Institute of Biotechnology, Universidad Politécnica de CartagenaCartagena, Spain; ^2^Department of Biology, Wake Forest University, Winston-SalemNC, USA; ^3^Department of Biochemistry, Purdue University, West LafayetteIN, USA

**Keywords:** floral scent, flower development, anthesis, phylogeny, biodiversity, chemodiversity, *Antirrhinum*

## Abstract

The genus *Antirrhinum* comprises about 28 species with a center of origin in the Iberian Peninsula. They show an important diversity of growing niches. We have performed a comprehensive analysis of scent profiles in eight wild species, *Antirrhinum linkianum, A. tortuosum, A. cirrigherum, A. latifolium, A. meonanthum, A. braun-blanquetii, A. barrelieri*, and *A. graniticum*. We used also two laboratory inbred lines *A. majus*, 165E and Sippe50. We identified 63 volatile organic compounds (VOCs) belonging to phenylpropanoids, benzenoids, mono- and sesquiterpenes, nitrogen-containing compounds, and aliphatic alcohols previously described in plants. Twenty-four VOCs were produced at levels higher than 2% of total VOC emission, while other VOCs were emitted in trace amounts. The absolute scent emission varied during flower maturation and species. The lowest emitting was *A. meonanthum* while *A. tortuosum* had the largest emissions. Species were clustered according to their scent profiles and the resulting dendrogram matched the current species phylogeny. However, two accessions, *A. majus* Sippe 50 and *A. braun-blanquetii*, showed development-specific changes in their VOC composition, suggesting a precise control and fine tuning of scent profiles. Cluster analysis of the different scent components failed to identify a specific synthesis pathway, indicating a key role of scent profiles as blends. There is considerable degree of chemodiversity in scent profiles in *Antirrhinum*. The specific developmental stage plays an important role in scent quantitative emissions. The relative robustness of the bouquets could be an adaptation to local pollinators.

## Introduction

The interaction between plants and other organisms is thought to be mediated by a complex set of traits among which the emission of chemical compounds plays a key role. The so-called plant volatiles are one of the most diverse set of molecules. Plant volatile emission can be classified according to the source of emission, i.e., leaves, flowers, and roots. And it can also be the result of certain reactions such as defense against herbivores or parasites. The emission of scent by flowers is a cue that helps to make floral sexual organs attractive to potential pollinators, but also works in parasite deterrence (Schiestl, 2010). In most flowers, floral scent is emitted by petals and stamens (Dudareva et al., 1996; Verdonk et al., 2003; Scalliet et al., 2006). Although over 1700 volatile organic compounds (VOCs) are described in plants, the actual composition of floral scent is not fully explored in most plant species (Knudsen et al., 2006).

Petal and stamen development in *Antirrhinum* and many other species is directly controlled by B function organ-identity genes (Egea Gutierrez-Cortines and Davies, 2000; Causier et al., 2010). The B function genes in *Antirrhinum* are the MADS-Box genes *DEFICIENS* and *GLOBOSA*. Their expression is required in a quantitative manner to attain fully developed petals and stamens (Bey et al., 2004; Manchado-Rojo et al., 2012). Floral scent emission is a late process starting shortly before anthesis in a variety of species (Knudsen et al., 2006), but its quantitative levels are regulated upstream by the B-function genes (Manchado-Rojo et al., 2012). Scent production varies after anthesis showing an increase in production till a point when sharp decreases are caused by flower aging and/or pollination (Pichersky et al., 1994; Ruiz-Ramon et al., 2014).

*Antirrhinum*, a genus native to the western Mediterranean region, comprises a monophyletic group with approx. 28 species (Liberal et al., 2014), traditionally assigned to the three morphological subsections or clades: *Kicksiella, Antirrhinum*, and *Streptosepalum* (Rothmaler, 1956; Webb, 1971; Sutton, 1988). The *Antirrhinum* flower has an occluded corolla (Vargas et al., 2010; Guzmán et al., 2015). It is apparently specialized in bee pollination as bees such as *Rhodanthidium sticticum* is the main pollinator of *A. microphyllum*, (Torres et al., 2003), and seven types of bees account for over 90% of the pollination visits in *Antirrhinum charidemi, Antirrhinum graniticum*, and *Antirrhinum braun-blanquetii* (Vargas et al., 2010). Despite the diversity the composition of the *Antirrhinum* genus floral scent, like that of many other plants, is basically unexplored and only *A. majus sp pseudomajus* and *A. striatum* have been analyzed with detail (Suchet et al., 2010).

In this work, we present a comprehensive analysis of floral VOCs in eight wild *Antirrhinum* species: *Antirrhinum linkianum, A. tortuosum, A. cirrigherum, A. latifolium, A. meonanthum, A. braun-blanquetii, A. barrelieri*, and *A. graniticum*. We have also used two laboratory inbred lines, *A. majus* 165E and Sippe50. These lines have been used for genetic studies, development of an *Antirrhinum majus* genetic map and for genetic transformation (Schwarz-Sommer et al., 2003, 2010; Manchado-Rojo et al., 2012, 2014). We identified at least 63 VOCs produced at one stage after anthesis and before petal senescence. Each species had a unique blend of VOCs, and tended to show a robust profile except for two species. The scent profiles allowed a cluster reconstruction that matched published phylogenies based on molecular markers indicating a uniqueness of scent signature for each species that may have implications for local adaptation.

## Materials and Methods

### Plant Material and Growth Conditions

**FIGURE 1:**
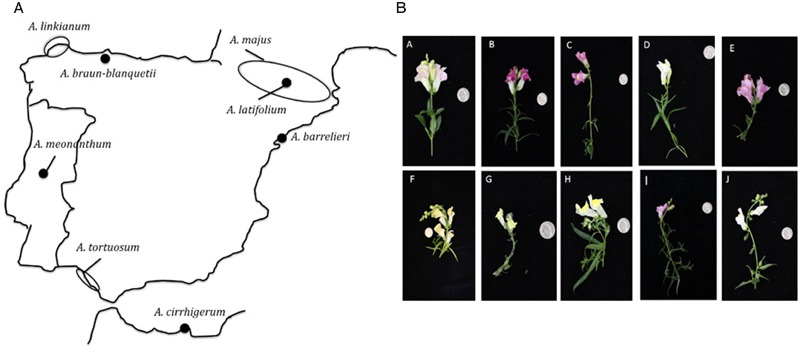
**Origin of *Antirhinum* species used in the study. (A)** Origin of seeds (filled circles) used in this study. For seeds of unknown origin, the area of distribution according to Vargas et al. (2009) is indicated (empty circle). *A. graniticum* is widely spread over central Iberian Penisula and the Western coast area, but absent in the North and East of the Iberian Peninsula (Vargas et al., 2010). **(B)** Flowers of the studied *Antirrhinum* species. A 25 cent USA-dollar is photographed for scale: (A) *A. majus* line 165E, (B) *A. majus* line Sippe 50, (C) *A. linkianum*, (D) *A. tortuosum*, (E) *A. cirrigherum*, (F) *A. latifolium*, (G) *A. meonanthum*, (H) *A. braun-blanquetii*, (I) *A. barrelieri*, and (J) *A. graniticum*.

We obtained eight wild species of *Antirrhinum* and two laboratory inbred lines (**Table 1**). The wild species include species of subsection *Antirrhinum*, series *Majora: A. barrelieri, A. cirrhigerum, A. graniticum, A. latifolium*, and *A. tortuosum* (Mateu-Andres and De Paco, 2005) as well as the two only members of subsection *Streptosepalum, A. braun-blanquetii* and *A. meonanthum* (Feng et al., 2009) (**Figure 1**). We also used two laboratory inbred lines, *A. majus* Sippe50 isolated at the beginning of the 20th century in Germany (Stubbe, 1966) and *A. majus* 165E developed at the John Innes Centre (Harrison and Carpenter, 1979; Sommer and Saedler, 1986). The geographical distribution of the species surveyed includes the Pyrenees, northern Spanish coast, Portugal, southern Spanish coast, and northern Africa (**Figure 1**). Plants were grown under standard greenhouse conditions using large pots of 3–5 l to increase the number of flowers obtained (Weiss et al., 2016). Four to five plants for each species and line were propagated and flowers were sampled randomly from these plants for further analysis.

**Table 1:** Name and origin/supply of *Antirrhinum* species.

Species name	Origin
*Antirrhinum barrelieri* Boreau	Vendrell, Tarragona Province, and Spain
*Antirrhinum braun-blanquetii* Rothm.	Province of Oviedo, Picos de Europa, and Spain
*Antirrhinum meonanthum* Hoffmanns and Link	Penacova and Portugal
*Antirrhinum latifolium* Mill.	Ville Franche, Pyrenees, and France
*Antirrhinum graniticum* Rothm.	Unknown
*Antirrhinum. linkianum*	Supplied by Bot. Garden, University of Coimbra, Portugal
*Antirrhinum cirrhigerum*	Unknown, Spain
*Antirrhinum tortuosum*	Unknown, Spain
**Laboratory lines**	
*Antirrhinum majus L.* line 165E	Our stocks
*A. majus L.* line Sippe 50	Supplied by IPK Gatersleben

### VOC Collection

Flower samples were taken daily during six days after flower opening and emitted volatiles were analyzed by dynamic headspace analysis (Raguso and Pellmyr, 1998). For each flower developmental stage, three randomly chosen, detached flowers were placed in 5% sucrose solution in transparent glass containers. Volatile sampling was performed over a 24-h period in a growth chamber (model E8; Conviron, Asheville, NC, USA) with a photoperiod of 12:12 light: dark conditions. Scent components were trapped with Porapak Q-filled glass syringes in a closed-loop scent collection system. Trapped volatiles were eluted from the adsorbent with dichloromethane.

### Gas-Chromatography Mass Spectrometry

Trapped floral volatiles were analyzed by gas chromatography–mass spectrometry (GC-MS) as described (Dudareva et al., 2003). Data analysis and volatile identification was performed with the MSD ChemStation (Agilent Technologies) software. The compounds were identified by comparing mass spectra and retention time (RT) data with those of authentic standards for benzaldehyde, β-myrcene, 2-ethyl-1 hexanol, β-ocimene, acetophenone, methyl benzoate, linalool, and methyl cinnamate, supplemented with information from the NIST11 spectral library. The relative contribution of volatile compounds was calculated based on the integrated area of particular peaks relative to the total integrated peak area for the flower opening stages I = day 1, II = day 3, and III = day 5. Total volatile amount was calculated based on integrated peak area of a defined amount of the internal standard naphthalene. Total amounts are given as integrated area of peaks normalized to naphthalene/g fresh weight (FW)^-1^ 24 h^-1^. Supplementary Figure S1 shows one chromatogram of each species at stage III. The different volatiles in percentages can be found Supplementary Table S1.

### Cluster Analysis and Principle Component Analysis of Volatiles

For hierarchical cluster analysis, the relative amounts of the 24 most abundant, major volatile compounds were used. We considered as major compounds those that accounted for equal or more than 2% of total amounts in the different flowering stages of the species or subspecies analyzed. The cluster analysis included the volatile profiles of flower opening stages I, II, and III as mentioned above. Clustering of species and developmental stages was achieved using R version 2.13.1, with Pearson correlation and average linkage serving as correlation and agglomeration methods.

Principal component analysis (PCA) was performed with absolute amounts of all VOCs (**Table 2**). Each sample collected was included in this analysis. To satisfy the assumption of linearity, absolute amounts were log10(*n*+1)-transformed prior to PCA. PCA with varimax rotation was performed with the prcomp command in the stats package in R version 2.13.1.

**Table 2:** List of volatile organic compounds (VOCs) identified in *Antirrhinum* and known to be biosynthesised by plants.

Plant emitted volatiles	CAS number	Retention time (RT)	% Probability
**Benzenoid – Aldehydes**			
Benzeneacetaldehyde	122-78-1	10.925	90
Benzaldehyde	100-52-7	9.9076	94
Benzaldehyde, 3-ethyl-	34246-54-3	13.316	95
Vanillin	121-33-5	17.488	90
Benzaldehyde, 4-ethyl-	4748-78-1	13.311	90
3,5-Dimethoxybenzaldehyde	7311-34-4	18.077	98
Benzaldehyde, 4-methoxy-	123-11-5	15.016	94
**Benzenoid – Ketones**			
Acetophenone	98-86-2	11.491	97
4-Acetylanisole	100-06-1	16.721	94
Ethanone, 1-(4-ethylphenyl)-	937-30-4	15.502	97
**Benzenoid – Esters**			
Benzyl Benzoate	120-51-4	22.924	98
Methyl benzoate	93-58-3	11.995	94
Benzoic acid, 3,5-dimethoxy-, methyl ester	2150-37-0	20.246	98
Methyl salicylate	119-36-8	13.934	97
Benzoic acid, 4-methoxy-, methyl ester	121-98-2	17.081	81
Benzoic acid, 2-butoxy-, methyl ester	606-45-1	13.934	97
**Benzenoid – Ethers**			
3,5-Dimethoxytoluene	4179-19-5	15.222	98
1,2,4-Trimethoxybenzene	135-77-3	16.973	94
Anisol	100-66-3	8.086	91
Benzene, 1,3,5-trimethoxy-	621-23-8	17.625	96
**Benzenoids – Benzenes**			
Benzene, 1,3-diethyl-	141-93-5	1.033	97
Benzene, 1,4-diethyl-	105-05-5	11.176	97
Benzene, 1,2-diethyl-	135-01-3	11.291	96
p-Xylene	106-42-3	6.959	95
Ethylbenzene	100-41-4	6.776	94
Benzene, 1,2,3-trimethyl-	526-73-8	9.826	92
Benzene, 1,4-dimethoxy-	150-78-7	13.294	96
Benzene, 1,2-dimethoxy-4-(2-propenyl)-	93-15-2	17.505	98
**Benzenoids – Alcohols**			
Benzyl Alcohol	100-51-6	10.679	95
3-Methoxy-5-methylphenol	3209-13-0	16.120	94
Cinnamyl alcohol	104-54-1	15.891	98
Benzenepropanol	122-97-4	14.547	98
Benzenemethanol, 4-methoxy-	105-13-5	15.502	95
Phenol, 4-(1,1-dimethylethyl)-2-methyl-	98-27-1	14.775	93
Phenol	108-95-2	9.540	74
**Isoprenoids–Monoterpenes**			
Myrcene	123-35-3	9.786	96
Ocimene	3779-61-1	11.002	98
Neo-allo-ocimene	7216-56-0	12.618	96
Linalool	78-70-6	12.058	97
Limonene	138-86-3	10.581	99
α-Pinene	80-56-8	8.476	95
Terpineol	98-55-5	13.849	90
**Isoprenoids–Sesquiterpenes**			
α-Farnesene	502-61-4	19.164	97
Nerolidol	7212-44-4	20.023	95
**Phenylpropanoids – Alcohols**			
Eugenol	97-53-0	16.784	98
**Phenylpropanoids – Esters**			
Methyl cinnamate	103-26-4	17.219	97
Cinnamyl formate	21040-45-9	16.692	98
**Phenylpropanoids –Aldehydes**			
Cinnamaldehyde	104-55-2	15.308	98
**Fatty acid derivatives – Aldehydes**			
Decanal	112-31-2	14.066	91
Nonanal	124-19-6	12.126	87
Hexanal, 2-ethyl-	123-05-7	8.951	81
**Fatty acid derivatives – Ketones**			
2-Pentadecanone, 6,10,14-trimethyl-	502-69-2	23.833	99
5-Hepten-2-one, 6-methyl- Methylheptenone	110-93-0	9.677	94
γ-Hexenol	928-96-1	6.656	91
**Fatty acid derivatives – Alcohols**			
1-Hexanol, 2-Ethyl,	104-76-7	10.576	90
Phenoxyethanol	122-99-6	14.346	95
**Fatty acid derivatives – Acids**			
Dodecanoic acid	143-07-7	19.885	91
**Amines and other nitrogen containing compounds**			
Indolizine	274-40-8	15.714	86
Indole	120-72-9	15.708	95
Methyl nicotinate	93-60-7	12.807	95
Benzyl nitrile	140-29-4	12.836	96
Diphenylamine	122-39-4	20.915	90
**Non-classified**			
1,3,5,7-Cyclooctatetraene	629-20-9	7.474	70

*Retention times are approximated and consistent between all the chromatograms analyzed. % Probability column indicates the existing chances of success in identifying compounds for a given RT, according to the NIST11 database. Internal standard (Naphthalene, CAS no. 91-20-3) probability is over 95%.*

## Results

### Phenotypic Space of Scent Emission

The emission of floral volatiles starts at late stages of petal morphogenesis requiring fully developed petals and anthesis (Manchado-Rojo et al., 2012; Muhlemann et al., 2012). We investigated the production of floral scent over a time span of six days after flower opening and identified a total of 63 based on NIST 11. There were 63 that matched VOCs previously identified in plants (**Table 2**). They belonged to the following chemical categories: phenylpropanoids, benzenoids, mono- and sesquiterpenes, nitrogen-containing compounds, and aliphatic alcohols (Knudsen et al., 1993). Amongst the compounds identified and found in a variety of plants and in *Antirrhinum* were benzenoids such as vanillin, o-acetanisole, methyl salicylate, anisole or cuminyl alcohol; isoprenes such as alpha pinene or terpineol. Phenylpropanoids included cinnamyl formate; fatty acid derivatives as aldehydes including octanal, decanal, nonanal or alcohols such as octanol or as acids. Flowers also emitted amines or nitrogen containing compounds such as methyl nicotinate, indole or indolicine 1,3,5,7-cyclooctatetraene, a non-classified compound.

We additionally found nine VOCs that had not been described previously as emitted by plant tissues (**Table 3**). They could be grouped into the classic set of benzenoids, phenylpropanoids, and fatty acid derivatives VOCs.

**Table 3:** List of new VOCs identified in *Antirrhinum* and previously unidentified in plants.

New volátiles	CAS number	RT	Quality
**Benzenoid – Ketones**			
Acetophenone, 2′-hydroxy-	118-93-4	13.311	97
**Benzenoid – Esters**			
Benzenepropanoic acid, methyl ester	103-25-3	15.731	92
**Benzenoids – Benzenes**			
Benzene, 1-(1,1-dimethylethyl)-4-methoxy-	5396-38-3	14.775	94
Benzene, 1-ethenyl-3-ethyl-	7525-62-4	11.766	96
**Benzenoids – Alcohols**			
Phenol, p-tert -butyl-	98-54-4	15.645	97
Phenol, 2,6-dimethoxy-4-(2-propenyl)-	6627-88-9	20.606	97
Benzenethanol	60-12-8	12.338	93
**Phenylpropanoids – Esters**			
Cinnamyl acetate	103-54-8	18.180	97
**Fatty acid derivatives – Alkanes**			
Adamantane, 1,3-dimethyl-	702-79-4	12.378	90

*Retention times are approximated and consistent between all the chromatograms analyzed. % Probability column indicates the existing chances of success in identifying compounds for a given RT, according to the NIST11 database. Internal standard (Naphthalene, CAS no. 91-20-3) probability is over 95%.*

**FIGURE 2:**
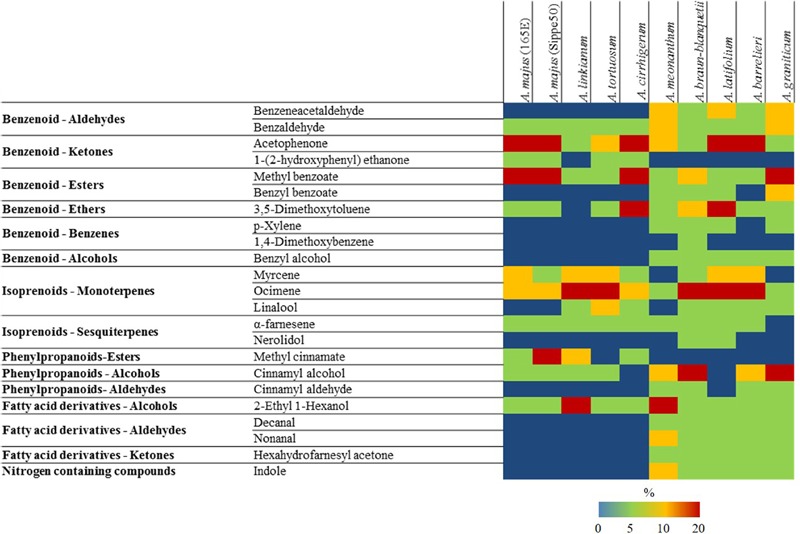
**Heat map with major VOCs (above 2% of total volatiles present in chromatograms) emitted by different species of *Antirrhinum*.** Colors reflect the maximum level of emission (%) in stages I–III for each species. Key color emission: blue (0%), green (<5%), yellow (<20%), and red (>20%).

From the large dataset presented, there were 24 major compounds comprising more than 2% of the scent emission in the different species (**Figure 2** and Supplementary Figure S1). Among these we found benzaldehyde, acetophenone, ocimene, and 2-ethyl 1-hexanol in all the species analyzed, comprising very different percentages of the scent profile. At the other side of the spectrum, 1,4-dimethoxybenzene was present only in *A. braun-blanquetii*, nerolidol was found in *A. braun-blanquetii* and *A. latifolium* and 5,9-dodecadien-2-one, 6,10-dimethyl was found in *A. meonanthum* and *A. graniticum*.

The complexity of the different suggested scent profiles in terms of number of VOCs emitted varied greatly. The most complex profile was exhibited by *A. braun-blanquetii* comprising 21 VOC compounds above 2% over at least one of the three developmental stages analyzed (**Figure 2** and Supplementary Figure S1). In contrast there were five accessions with a much simpler scent profile such as *A. linkianum* with 10 major VOCs followed by *A. cirrhigerum, A. majus* Sippe50 and *A. tortuosum* with 11 VOCs and *A. majus* 165E with 12.

In summary, the species analyzed appeared to show two distinct suggested profiles as those with relative low scent complexity lack irregular terpenes, fatty acid aldehydes and ketones, and nitrogen containing compounds.

### Changes in Total and Relative Emission of VOCs during Flower Development

**FIGURE 3:**
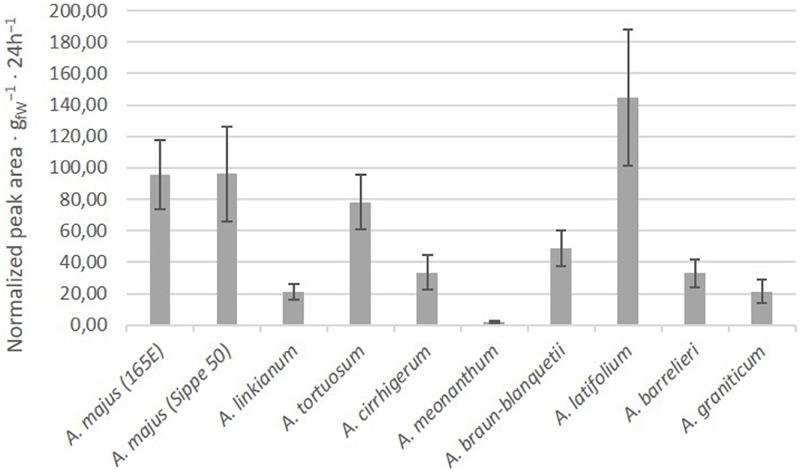
**Average emission of VOCs in the different accessions.** Quantities are total integrated area normalized to naphthalene/g 24 h^-1^. Error bars correspond to standard error.

We analyzed the scent emission during a period of seven days. Flowers of all species produced scent during the entire sampling period. The average emission of scent during the period varied between species (**Figure 3**). The lowest emitting species was *A. meonanthum* while the species with larger levels of production corresponded to *A. latifolium* followed by the two *A. majus* 165E and Sippe50 inbred lines.

**FIGURE 4:**
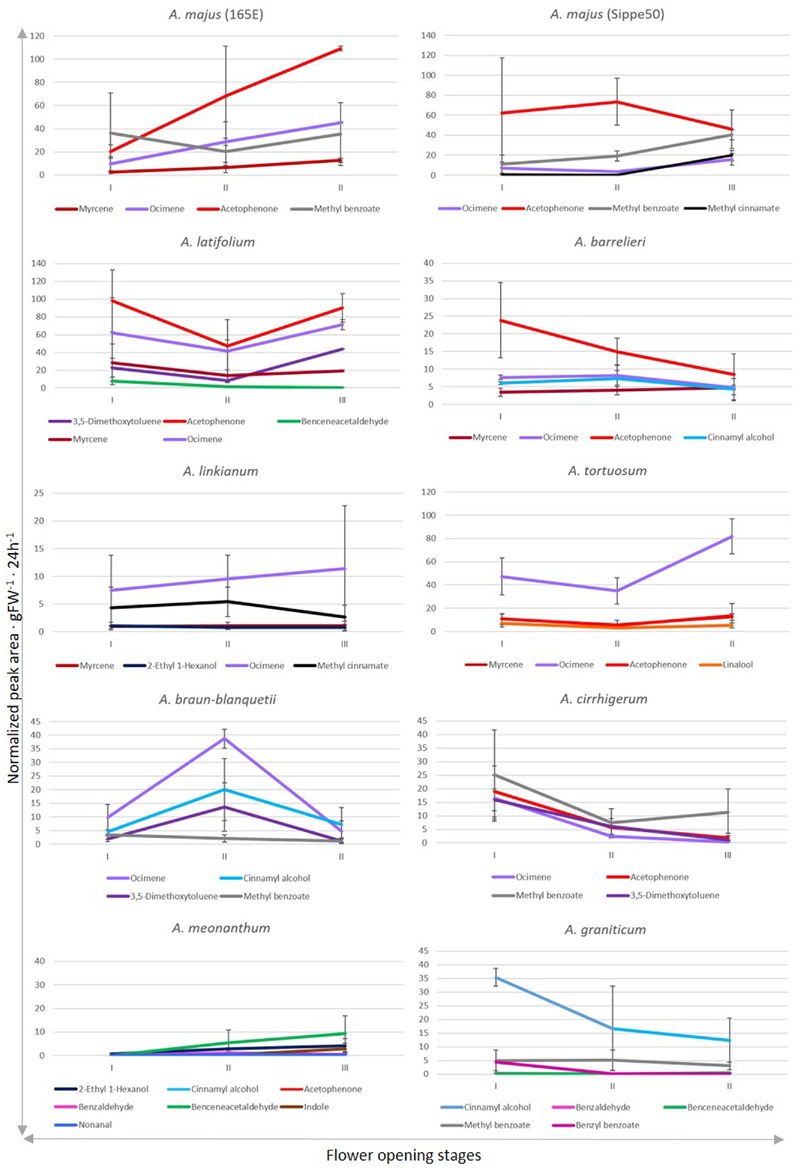
**Changes in emission of selected VOCs after anthesis.** Stages of development described as stage I, II, and III correspond to 1–2, 3–4, and 5–6 days after anthesis. Quantities are reflected as total peak integrated areas normalized to naphthalene/g 24 h^-1^. Error bars correspond to standard error. The VOCs represented here have an emission above 5% of the total amount of emitted VOCs.

We analyzed the quantitative changes in emission of the different compounds throughout development (**Figure 4**). The two *A. majus* inbred lines used for many experiments in plant development and the wild species *A. latifolium* and *A. barrelieri* produced acetophenone as major volatile. They also showed comparable levels of methyl benzoate and ocimene emission. However, they differed in the emission of myrcene *by A. majus 165E* and methyl cinnamate by *A. majus S.50*. The profile of *A. latifolium* included ocimene, 3,5-dimethoxytoluene, benzeneacetaldehyde, and myrcene, while *A. barrelieri* emitted ocimene, cinnamyl alcohol, and myrcene.

There were three species, *A. linkianum, A. tortuosum, and A. braun-blanquetii* that emitted ocimene as major volatile. However, the rest of the volatiles were not in common as *A. linkianum* produced methyl cinnamate, myrcene, and 2-ethyl 1-hexanol. The scent profile of *A. tortuosum* included myrcene, acetophenone, and linalool while *A. braun-blanquetii* showed high levels of cinnamyl alcohol, 3,5-dimethoxytoluene, and methyl benzoate.

Finally, three species showed a different VOC as major compound. The major VOC in *A. cirrhigerum* was methyl benzoate, and emitted acetophenone, 3,5-dimethoxytoluene and ocimene. The scent profile of *A. meonanthum* was complex as its debut was dominated by 2-ethyl-hexanol but was taken over by benzene acetaldehyde. It also emitted acetophenone, cinnamyl alcohol, benzaldehyde, nonanal, and the nitrogen containing indole. The main component emitted by *A. graniticum* was cinnamyl alcohol, methyl benzoate, benzyl benzoate, benzaldehyde, and benzeneacetaldehyde.

Concerning the quantitative changes in emission during flower maturation, the quantities varied and the variance was high. This is probably due to temperature changes during flower maturation. Thus a general pattern of emission cannot be found for all the species.

### Scent-Based Clustering of *Antirrhinum* Species and Robustness of Scent Profiles

**FIGURE 5:**
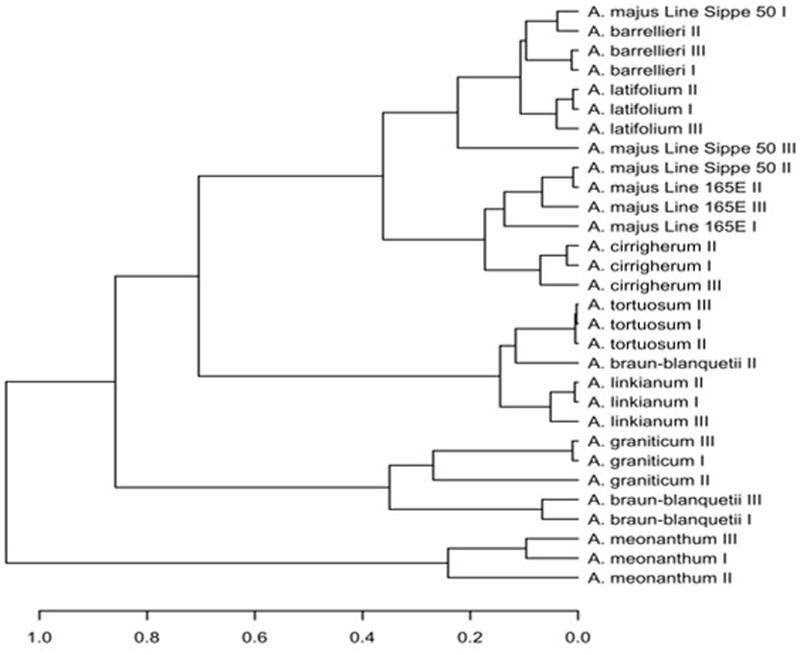
**Cluster analysis based on scent profiles of the different *Antirrhinum* species sampled at different flower developmental stages**.

To determine whether differences in scent emission between species are greater than emission differences between developmental stages within species, we collected volatile samples for the developmental stages I–III. Suggested volatile profiles for most of the species presented here, except *A. majus* line Sippe 50 and *A. braun-blanquetii*, clustered together for all flower developmental stages (**Figure 5**), demonstrating that the profile of the 24 major volatiles changed less between developmental stages than between the species. In case of *A. majus* Sippe 50 and A. *braun-blanquetii*, suggested scent profiles of different flower ages clustered in different branches, indicating variations in the composition of fragrances during development. This highlights that sampling several developmental stages is a critical factor if the volatile profile is to be used for taxonomic interpretation.

The suggested volatile profiles of *A. meonanthum and A. braun-blanquetii*, both belonging to subsection *Streptosepalum*, build separate clusters from members of subsection *Antirrhinum*, with the exception of *A. graniticum*, which clustered with *A. braun-blanquetii*, separate from all other members of subsection *Antirrhinum*.

Within subsection *Antirrhinum*, except for *A. graniticum*, species branched into two main clusters. One of these two branches contained *A. linkianum* and *tortuosum*. Within the second major branch, *A. majus* and *cirrigherum* on one side and *A. barrilieri* and *latifolium* on the other side showed a closer relatedness.

### Identification of Associated Odor Descriptors by PCA

To identify scent compounds that contribute to the variation in VOC profiles between species, we performed a PCA. We extracted four components that account for 82% of the variance in the data (**Table 4**). The first principal component, which explains 58% of the variance observed in scent emission between species, displays negative loadings for acetophenone and ocimene. The two compounds with the highest correlation to the second principal component were cinnamyl alcohol and 2-ethyl-1-hexanol. The third principal component contrasted the presence of cinnamyl alcohol with that of acetophenone, with a positive loading for cinnamyl alcohol and a negative loading for acetophenone. Lastly, the fourth principal component was highly correlated to methyl benzoate and ocimene. These data reveal that variance in volatile profiles between *Antirrhinum* species is caused by differences in emission levels of VOCs originating from different biosynthetic pathways, rather than by the presence of VOCs derived from a single pathway within a species. This observation suggests a selection for complex profiles rather than for a specific pathway.

**Table 4:** Principal component loadings for the four principal components explaining more than 80% of the variance.

Compound	PC1 (58.32%)	PC2 (10.90%)	PC3 (7.20%)	PC4 (5.89%)
Benzaldehyde	-0.158	-0.245	-0.118	0.064
Benzeneacetaldehyde	-0.113	-0.227	-0.156	0.079
Methyl benzoate	-0.328	-0.150	0.089	-**0.659**
Cinnamaldehyde	-0.043	-0.151	0.153	-0.062
Cinnamyl alcohol	-0.226	-**0.469**	**0.589**	-0.081
Methyl cinnamate	-0.111	-0.147	-0.170	0.109
Acetophenone	-**0.487**	0.316	-**0.395**	-0.396
3,5-Dimethoxytoluene	-0.271	-0.042	0.147	0.060
Indole	-0.090	-0.284	-0.099	0.061
1-Hexanol, 2-ethyl-	-0.191	-**0.367**	-0.381	0.214
Nonanal	-0.059	-0.169	-0.176	0.063
a,β-Ocimene	-**0.537**	0.277	0.235	**0.423**
β-Myrcene	-0.310	0.252	0.034	0.187
α-Farnesene	-0.061	0.003	-0.065	0.155

*The variance explained by each component is indicated in parentheses. Only compounds with a loading ≥| 0.15| in at least one component are shown. Loadings with highest correlation to individual components are in boldface.*

**FIGURE 6:**
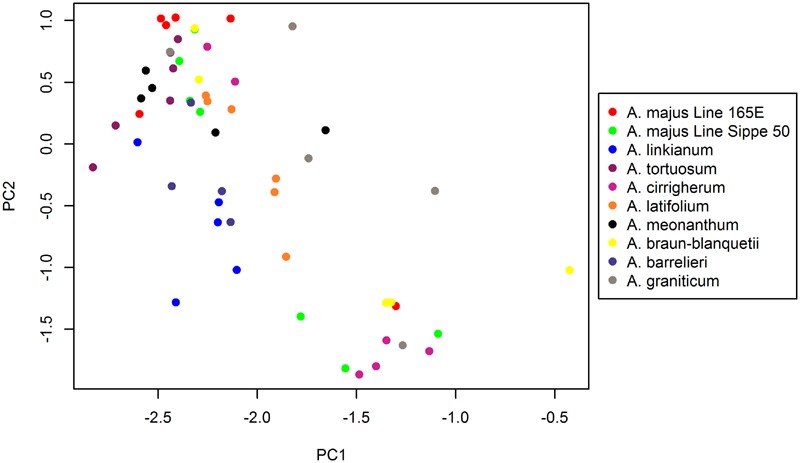
**Principal component analysis (PCA) of *Antirrhinum* species based on emitted volatiles.** The two axes represent principal components 1 and 2, which explain 58.32% and 10.90% of the total variance, respectively.

By plotting the principal component scores of each species (**Figure 6**), we found that the scores for each species along the first and second principal component (PC1 and PC2) axis most display a considerable spread. *A. braun-blanquetii* and *A. majus* Line Sippe 50, for example, have a large variation in scores along PC1 and PC2, reflecting findings from the cluster analysis. Indeed, developmental stages for these species did not cluster as tightly as for other species.

## Discussion

In the present study, we have determined the phenotypic space of scent profiles in eight wild species of *Antirrhinum* and two laboratory inbred lines. The species used in the present study are found in very distant regions of the Iberian Peninsula and have very different ecological niches. Our data show that the complexity of scent profiles in the *Antirrhinum* genus is remarkable with at least 63 different compounds previously identified, and an additional set of nine that may require further studies to verify their presence in plants. The number of independent major VOCs found is similar to most species described. Species have been identified with as little as one compound emitted by *Nicotiana africana* to 35 in *N. bonariensis* during the night (Raguso et al., 2006). Other species such as Petunia (Kondo et al., 2007) have a range of scent components between 10 and 21, similar to the one found in the current study. The diversity of compounds produced in *Antirrhinum* is large but 500 VOCs have been described in studies in roses demonstrating the possibility of being a highly complex trait (Knudsen et al., 2006).

The most common volatiles found in between 71 and 52% of all plant families are, in decreasing order, limonene, ocimene, myrcene, linalool alpha-pinene, benzaldehyde, ß-pinene, methyl 2-hydroxybenzoate also known as methyl salicylate, benzyl alcohol, 2-phenyl ethanol, caryophyllene, and 6-methyl-5-hepten-2-one (Knudsen et al., 2006). The compounds found in most *Antirrhinum* species thus fall within the major VOCs found in flowering plants. The main VOCs found in all the *Antirrhinum* accessions analyzed were benzaldehyde, acetophenone ocimene and the fatty acid derivative 2-ethyl 1-hexanol indicating a common set of VOCs in the species analyzed. Highly ubiquitous compound such as benzyl alcohol was clearly forming a separate group of accessions that either did or did not produce this specific compound (**Figure 2**). The only common scent compound we did not find was caryophyllene (Knudsen et al., 2006), and others such as the commonly found limonene, or alpha and beta pinene were detected only in trace amounts. Other compounds found less often but still generally found in plants included indole. Altogether the major VOCs found in *Antirrhinum* are a good representation of the different biosynthetic pathways found for scent VOCs in the plant kingdom. This is in sharp contrast to well established models such as *Arabidopsis* that produces sesquiterpenes as major VOCs (Tholl et al., 2005), or Petunia producing mainly phenylpropanoids (Hoballah et al., 2007; Bombarely et al., 2016).

The scent profile of flowers of a specific plant can change in response to the physiological stage of the flower, including flower age (Pichersky et al., 1994; Dudareva et al., 1998), pollination status the circadian rhythm or temperature (Sagae et al., 2008; Cna’ani et al., 2014). We found that all the species analyzed except two, *A. meonanthum* and *A. latifolium*, displayed an increase in emission followed by a decrease after 5–6 days after anthesis. Moreover, *A. majus* Sippe 50 and *A. graniticum* also had a major VOC showing a trend increasing towards the end of the flower lifespan. As the compounds showing this trend were very diverse including acetophenone, ocimene, 2-ethyl hexanol, indole, cinnamyl alcohol, and methyl cinnamate, we cannot conclude that it is a single pathway that is differentially regulated during flower aging. Our results indicate that there must be a common mechanism of control involved in the quantitative control of scent emission linked to flower aging, and this mechanism is subject to changes as found for individual components that differed in the emission kinetics.

The diversity among the major compounds was strong enough to allow a phylogenetic reconstruction. There are several phylogenies described for the genus *Antirrhinum*, including reconstructions based on chloroplast genes such as combined *psbA-trnH/trnT-trnL/ trnK-matK/trnS-trnG* sequences (Carrio et al., 2010), *trnS-trnG/trnK-matK* (Liberal et al., 2014) statistical parsimony networks of plastid haplotypes *trnS-trnG* and *trnK-matK* (Vargas et al., 2009), the nuclear *CYCLOIDEA* gene (Gübitz et al., 2003), and AFLP nuclear markers (Wilson and Hudson, 2011). All the aforementioned studies show *A. meonanthum* and *A. braun-blanquetii* are on a single clade while the rest of the species analyzed in the current study cluster together. Our data, show clustering of *A. menonanthum* and *A. braun-blanquetii*, while the other species form a different clade. Thus we can conclude that the complex scent profiles and both chloroplast and nuclear markers show a similar separation. A current hypothesis is that a multilocus under selection pressure maybe responsible for the complex phylogeny of *Antirrhinum* (Wilson and Hudson, 2011). Indeed the major local pollinators have been analyzed for three different *Antirrhinum* species and they are different (Vargas et al., 2010). Amongst the species studied, two are present in our work, i.e., *A. braun-blanquetii* and *A. graniticum*. However, we do not have evidence about a co-evolution or selection of the different scent profiles found in the different species and local pollinators, and they could be the result of a combination of selection and genetic drift. Variation between the different species is not based on single pathways but appears to occur at the aroma level, i.e., at the level of combination of components. As the levels of monoterpenes in *A. meonanthum* and *A. graniticum* are nearly absent, it remains to be determined if these changes are the result of single mutations affecting regulatory elements or key enzymes in the biosynthesis pathway.

The number and type of VOCs found in the *Antirrhinum* species analyzed indicated that there are several biosynthetic pathways that in parallel give rise to the scent blends identified. An important question raised is if the different profiles identified are the result of differences in biosynthetic pathways or rather result from the combination of components. As compounds belonging to a single pathway maybe correlated they would obscure statistics. Our data show that this is not the case. The first two compounds accounting for 58% of the variance correspond to the benzenoid acetophenone and the terpenoid ocimene, indicating that the major compounds do not belong to single pathways. This was corroborated with the other compounds that showed significant effects shared by cinnamyl alcohol, a benzenoid and 1-hexanol-2-ethyl, a fatty acid derivative. Altogether the PCA analysis indicates that the different scent profiles identified are not the result of changes in regulatory pathways or changes in one specific type of scent compound, suggesting a scent structure based on blends in *Antirrhinum*. This is not always the case as scent profiles with major components belonging to distinct pathways have been identified (Majetic et al., 2007). Our results do not exclude the possibility of finding other species where changes in regulatory genes or key enzymes will cause changes in complete VOC biosynthesis pathways. Indeed the only *Antirrhinum* wild species described so far *A. majus. Ssp pseudomajus* and *A. majus ssp striatum* differ in the emission of three benzenoids (Suchet et al., 2010), indicating a complex scenario in terms of scent profiles and differences between species.

Our data show that in general the *Antirrhinum* genus tends to have a robust scent profile. The fact that *A. braun-blanquetii* and *A. majus* Sippe 50 display modified scent profiles with aging indicates a genetic component establishing the complete scent profile. In this case it is not the effect of a single master activator as scent was produced by both species. As a pathway of regulatory genes plays a key role in control of scent production in Petunia (Klahre et al., 2011; Van Moerkercke et al., 2011; Spitzer-Rimon et al., 2012), activation of floral scent production in *Antirrhinum* at anthesis may be controlled by several non-redundant genes. Our results also suggest that the use of scent profiles for phylogenetic analysis may require sampling at different ages or developmental stages in order to define profiles that may or may not resolve distances. The richness of volatiles and the marked differences between the different species open the possibility to study the genetic structure of scent as a trait, and its use in evolutionary studies. The robustness of scent profiles may be seen as a signature and it may help in creating fidelity to pollinators.

## Author Contributions

JW, JM, ND, and ME-C designed experiments; JW, obtained data; JW, JM, VR-H, and ME-C analyzed data; JW, JM, VR-H, ND, and ME-C wrote the manuscript.

## Conflict of Interest Statement

The authors declare that the research was conducted in the absence of any commercial or financial relationships that could be construed as a potential conflict of interest.
