# Supplementary material for: Phenotypic Space and Variation of Floral Scent Profiles during Late Flower Development in Antirrhinum
Source: Front Plant Sci. 2016 Dec 21;7:1903. doi: 10.3389/fpls.2016.01903 (PMC5174079; doi:10.3389/fpls.2016.01903)
Supplement: Supplementary file 1 [file Table_1.DOCX]

| Table S1. Percentage of major volatile compounds in *Antirrhinum majus* laboratory inbred lines and wild species (*A. linkianum, A. tortuosum, A. cirrhigerum, A. meonanthum, A. braun-blanquetii, A. latifolium, A. barrelieri a*nd *A. graniticum*). Percentage is based on volatile collection from three radomly chosen flowers at flower opening stages I = day 1 -2; II = day 3 -4; III day 5 -6. Volatiles that account for ≥1% of total compounds in at least two samples of one species are listed. Information of main flower colour included for each species. | | | | | | | | | | | | | | | |
| --- | --- | --- | --- | --- | --- | --- | --- | --- | --- | --- | --- | --- | --- | --- | --- |
|  | ***A. majus* line 165E** | | | ***A. majus* line Sippe 50** | | | ***A. linkianum*** | | | ***A. tortuosum*** | | | ***A. cirrhigerum*** | | |
| ***Flower colour*** | pink-white | | | magenta | | | magenta | | | yellow-white | | | magenta | | |
| ***Flower opening stage*** | I | II | III | I | II | III | I | II | III | I | II | III | I | II | III |
| **Benzenoid-Aldehydes** | | | | | | | | | | | | | | | |
| Benzaldehyde | 3.0 | 2.8 | 1.3 | 0.1 | 0.5 | 0.2 | 3.1 | 0.5 |  | 1.6 | 0.8 | 0.9 | 0.6 | 1.1 | 0.3 |
| **Benzenoid - Ketones** | | | | | | | | | | | | | | | |
| Acetophenone | 20.3 | 33.5 | 43.9 | 70.8 | 39.0 | 46.5 |  | 0.2 |  | 12.5 | 9.5 | 14.5 | 20.3 | 23.8 | 33.5 |
| 1-(2-hydroxyphenyl) ethanone | 0.6 | 0.7 | 0.9 | 1.1 |  | 0.4 |  |  |  | 0.3 |  |  | 1.6 | 1.1 | 0.6 |
| **Benzenoid - Esters** | | | | | | | | | | | | | | | |
| Methyl benzoate | 55.7 | 41.7 | 25.6 | 12.3 | 40.4 | 19.4 | 1.5 | 3.0 | 0.2 | 1.1 | 0.2 | 0.6 | 31.3 | 28.0 | 21.0 |
| **Benzenoid - Ethers** | | | | | | | | | | | | | | | |
| 3.5-Dimethoxytoluene | 0.9 | 0.2 | 0.3 | 0.1 |  |  |  |  |  | 2.1 | 1.4 | 4.0 | 17.3 | 22.7 | 18.9 |
| **Isoprenoids-Monoterpenes** | | | | | | | | | | | | | | | |
| Myrcene | 2.8 | 2.7 | 6.0 | 1.9 | 2.5 | 0.7 | 5.4 | 4.3 | 4.9 | 10.5 | 10.2 | 9.3 |  | 4.0 | 4.46 |
| Ocimene | 12.5 | 14.5 | 18.3 | 8.1 | 10.9 | 9.0 | 23.5 | 44.1 | 59.3 | 56.9 | 65.3 | 58.6 | 16.8 | 14.0 | 17.0 |
| Linalool |  |  |  |  |  |  |  | 0.3 | 0.7 | 7 | 5.7 | 4.6 |  | 1.4 |  |
| **Isoprenoids-Sesquiterpenes** | | | | | | | | | | | | | | | |
| α-Farnesene | 0.3 | 0.1 | 0.1 | 0.5 | 0.3 |  | 1.8 | 2.4 | 4 | 1.5 |  | 0.9 | 0.2 |  |  |
| **Phenylpropanoids -Esters** | | | | | | | | | | | | | | | |
| Methyl cinnamate | 0.5 | 0.2 | 0.4 | 0.1 | 0.2 | 21.4 | 13.7 | 22.7 | 12.4 |  |  |  | 0.5 | 0.2 | 1.6 |
| **Phenylpropanoids - Alcohols** | | | | | | | | | | | | | | | |
| Cinnamyl alcohol | 0.3 |  | 0.5 | 1.5 | 1.0 | 0.6 | 1.5 | 2.6 | 0.3 | 0.2 | 1.5 | 0.7 |  |  |  |
| **Fatty acid derivatives - Alcohols** | | | | | | | | | | | | | | | |
| 2-Ethyl 1-Hexanol |  | 0.5 | 0.9 | 0.5 | 1.0 | 0.4 | 20.5 | 3.8 | 3.6 | 2.2 | 0.3 | 0.6 | 2.2 | 0.6 | 0.8 |

|  | ***A. meonanthum*** | | | ***A. braun-blanquetii*** | | | ***A. latifolium*** | | | ***A. barrelieri*** | | | ***A. graniticum*** | | |
| --- | --- | --- | --- | --- | --- | --- | --- | --- | --- | --- | --- | --- | --- | --- | --- |
| ***Flower color*** | yellow | | | yellow | | | white | | | magenta | | | white-pink | | |
| ***Flower opening stage*** | I | II | III | I | II | III | I | II | III | I | II | III | I | II | III |
| **Benzenoid-Aldehydes** | | | | | | | | | | | | | | | |
| Benzeneacetaldehyde | 9.9 | 2.7 |  | 0.1 | 0.7 | 0.1 | 0.5 | 6.3 |  | 0.6 | 0.3 | 0.7 | 2.6 | 6.9 | 1.6 |
| Benzaldehyde | 8.2 | 10.0 | 3.0 | 2.0 | 1.8 | 1.3 | 0.7 | 2.0 | 0.1 | 0.6 | 0.2 | 0.7 | 2.6 | 6.9 | 1.6 |
| **Benzenoid - Ketones** | | | | | | | | | | | | | | | |
| Acetophenone | 2.5 | 8.7 | 5.0 | 0.3 |  | 0.2 | 49.0 | 21.4 | 32.8 | 33.5 | 57.9 | 38.1 | 1.0 |  | 1.0 |
| **Benzenoid - Esters** | | | | | | | | | | | | | | | |
| Methyl benzoate |  | 2.3 | 1.1 | 1.9 | 4.7 | 6.6 | 0.7 | 1.1 | 1.8 | 0.9 | 1.5 | 5.0 | 5.4 | 36.5 | 13 |
| Benzyl Benzoate | 4.2 | 1.0 |  |  | 0.25 |  | 0.1 | 0.8 |  |  |  |  | 0.6 | 9.4 | 0.3 |
| **Benzenoid - Ethers** | | | | | | | | | | | | | | | |
| 3.5-Dimethoxytoluene |  | 3.7 | 1.8 | 11.6 | 9.1 | 5.8 | 8.5 | 22.7 | 19.3 | 2.0 | 1.0 | 4.1 | 0.5 |  | 1.8 |
| **Benzenoids - Benzenes** | | | | | | | | | | | | | | | |
| p-Xylene | 0.2 | 0.4 |  | 4.0 | 5.3 |  |  | 0.1 | 0.1 |  |  |  | 0.5 |  |  |
| 1.4-Dimethoxybenzene |  |  |  | 1.0 | 1.1 | 1.0 |  |  |  |  |  |  |  |  |  |
| **Benzenoids - Alcohols** | | | | | | | | | | | | | | | |
| Benzyl Alcohol | 3.0 | 5.3 | 1.9 | 0.5 | 0.9 | 0.5 |  | 1.0 |  | 0.2 |  |  | 1.2 | 2.7 | 0.5 |
| **Isoprenoids-Monoterpenes** | | | | | | | | | | | | | | | |
| β-Myrcene |  |  |  | 3.2 | 2.8 | 0.1 | 9.9 | 18.0 | 8.0 | 5.8 | 7.8 | 9.4 |  |  |  |
| β-Ocimene (Z) |  |  | 0.3 | 50.1 | 44.0 | 25.6 | 24.8 | 14.8 | 28.96 | 17.9 | 13.8 | 19.9 | 1.2 |  |  |
| Linalool |  |  |  | 0.8 |  | 0.5 | 1.6 | 1.3 | 2.2 | 1.5 | 2.2 |  | 1.8 |  | 1.1 |
| **Isoprenoids-Sesquiterpenes** | | | | | | | | | | | | | | | |
| a-Farnesene | 2.2 | 1.2 |  |  | 0.1 |  | 0.3 | 0.1 | 0.4 | 0.3 | 1.0 | 0.2 |  |  |  |
| Nerolidol |  |  |  | 3.0 | 3.9 | 2.7 | 1.8 |  | 3.7 |  |  |  |  |  |  |
| **Phenylpropanoids - Alcohols** | | | | | | | | | | | | | | | |
| Cinnamyl alcohol |  | 8.0 |  | 19.0 | 23.6 | 40.1 |  |  |  | 14.2 | 10.8 | 17.4 | 65.2 | 32.5 | 60.7 |
| **Phenylpropanoids - Aldehydes** | | | | | | | | | | | | | | | |
| Cinnamyl aldehyde |  | 1.0 |  | 1.2 | 1.6 | 1.9 |  |  |  | 1.1 | 0.6 | 0.8 | 2.7 | 1.0 | 2.7 |
| **Fatty acid derivatives - Alcohols** | | | | | | | | | | | | | | | |
| 2-ethyl 1-Hexanol | 45.0 | 16.4 | 25.0 | 0.9 | 0.1 | 2.1 |  |  | 0.5 | 1.2 | 0.4 |  | 2.8 |  | 4.1 |
| **Fatty acid derivatives - Aldehydes** | | | | | | | | | | | | | | | |
| Decanal | 2.4 | 3.9 | 1.2 |  | 0.1 | 0.1 | 0.1 |  |  | 0.2 |  |  | 0.4 | 0.8 | 0.3 |
| Nonanal | 2.9 | 6.8 | 2.2 | 0.1 | 0.2 |  | 0.2 | 0.4 | 0.1 | 0.4 |  |  | 0.7 | 1.1 | 0.5 |
| **Fatty acid derivatives - Ketones** | | | | | | | | | | | | | | | |
| Hexahydrofarnesyl acetone | 2.9 | 2.1 | 1.5 | 0.3 | 0.2 | 0.5 | 0.3 | 2.0 | 0.4 |  | 0.2 |  | 1.0 |  | 1.2 |
| **Amines a other nitrogen containing compounds** | | | | | | | | | | | | | | | |
| Indole | 5.6 | 7.4 | 18.1 | 1.2 | 1.0 | 2.1 |  |  | 0.1 | 0.2 | 1.1 | 1.2 | 1.9 | 1.2 | 3.0 |
