# Supplementary material for: Phenotypic Space and Variation of Floral Scent Profiles during Late Flower Development in Antirrhinum
Source: Front Plant Sci. 2016 Dec 21;7:1903. doi: 10.3389/fpls.2016.01903 (PMC5174079; doi:10.3389/fpls.2016.01903)
Supplement: Supplementary file 2 [file Image_1.PDF]

Supplementary material

Figure S1. . Gas chromatograms of volatiles of *Antirrhinum*. Chromatograms correspond to stage III for all species. Peak areas are representative of emitted quantities, and the X axis corresponds to retention times. Major peaks are depicted and naphthalene is also shown as internal standard. The naphthalene peak corresponds to 100 nanograms

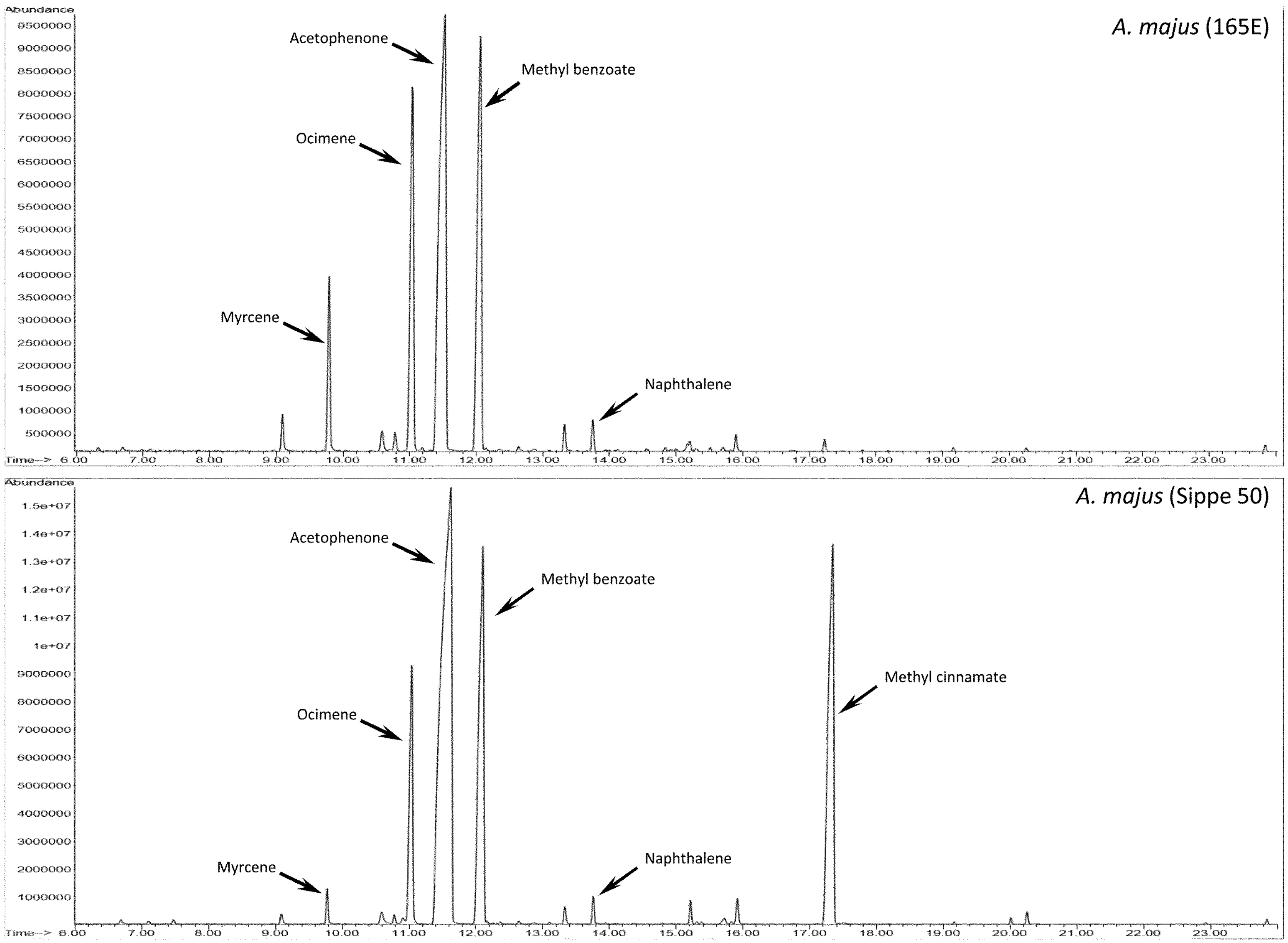

A. majus (165E)
Abundance
Acetophenone
Methyl benzoate
Ocimene
Myrcene
Naphthalene
Time--> 6.00 7.00 8.00 9.00 10.00 11.00 12.00 13.00 14.00 15.00 16.00 17.00 18.00 19.00 20.00 21.00 22.00 23.00
A. majus (Sippe 50)
Abundance
Acetophenone
Methyl benzoate
Methyl cinnamate
Ocimene
Myrcene
Naphthalene
Time--> 6.00 7.00 8.00 9.00 10.00 11.00 12.00 13.00 14.00 15.00 16.00 17.00 18.00 19.00 20.00 21.00 22.00 23.00

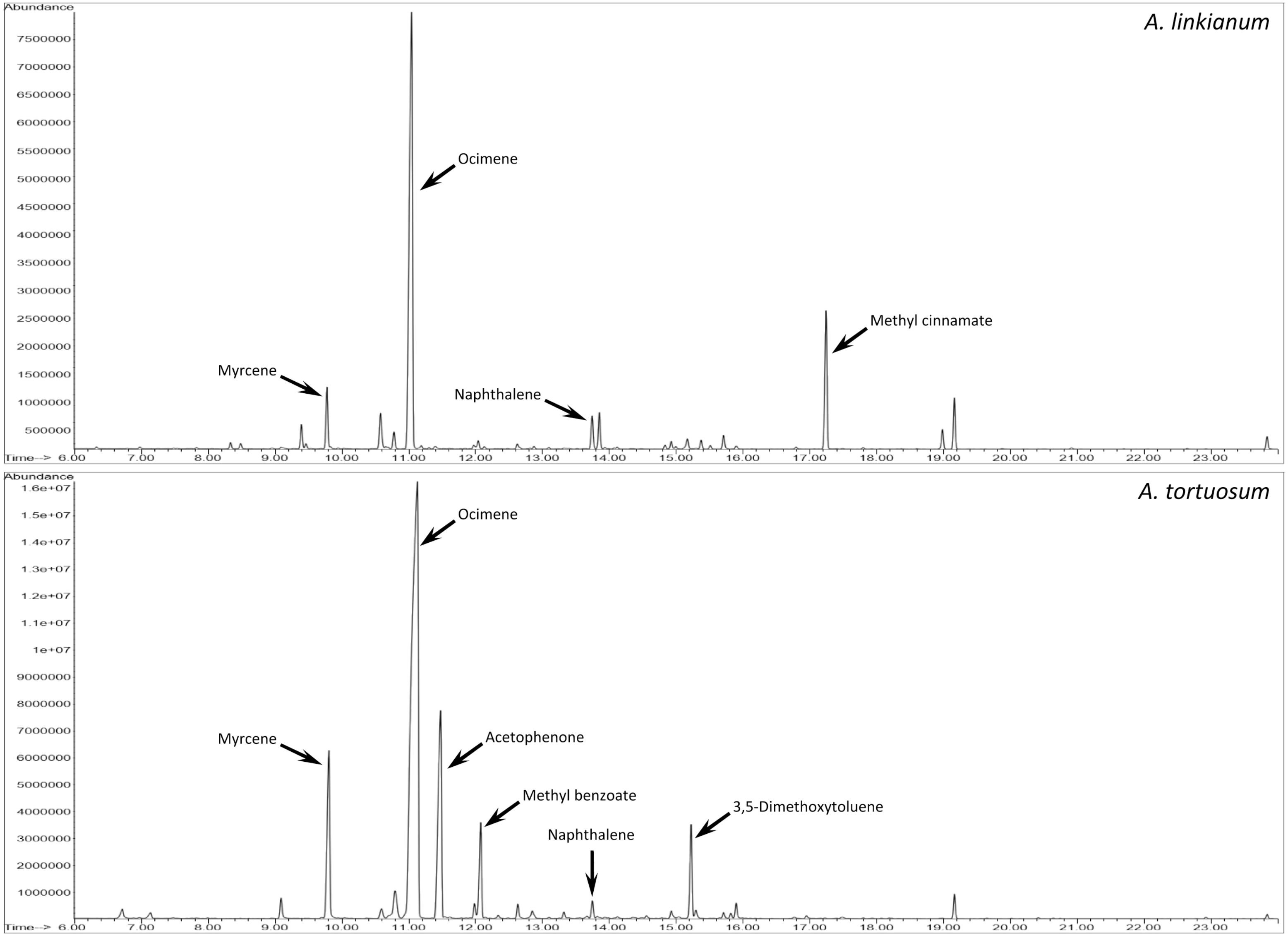
A. linkianum
Abundance
Time-->
Ocimene
Myrcene
Naphthalene
Methyl cinnamate
A. tortuosum
Abundance
Time-->
Ocimene
Myrcene
Acetophenone
Methyl benzoate
Naphthalene
3,5-Dimethoxytoluene

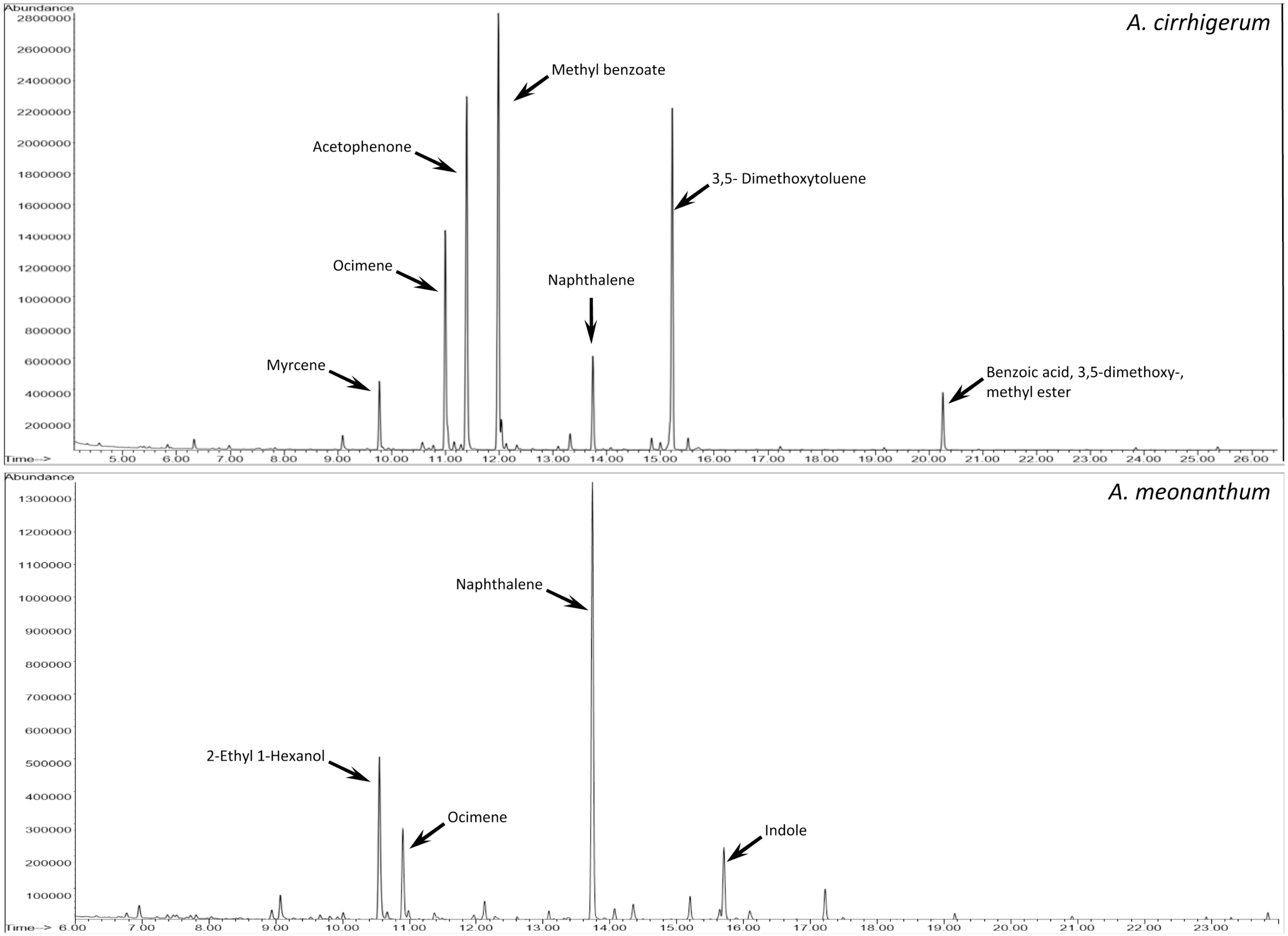

A. cirrhigerum
Abundance
Time-->
Methyl benzoate
Acetophenone
3,5- Dimethoxytoluene
Ocimene
Naphthalene
Myrcene
Benzoic acid, 3,5-dimethoxy-, methyl ester
A. meonanthum
Abundance
Naphthalene
2-Ethyl 1-Hexanol
Ocimene
Indole

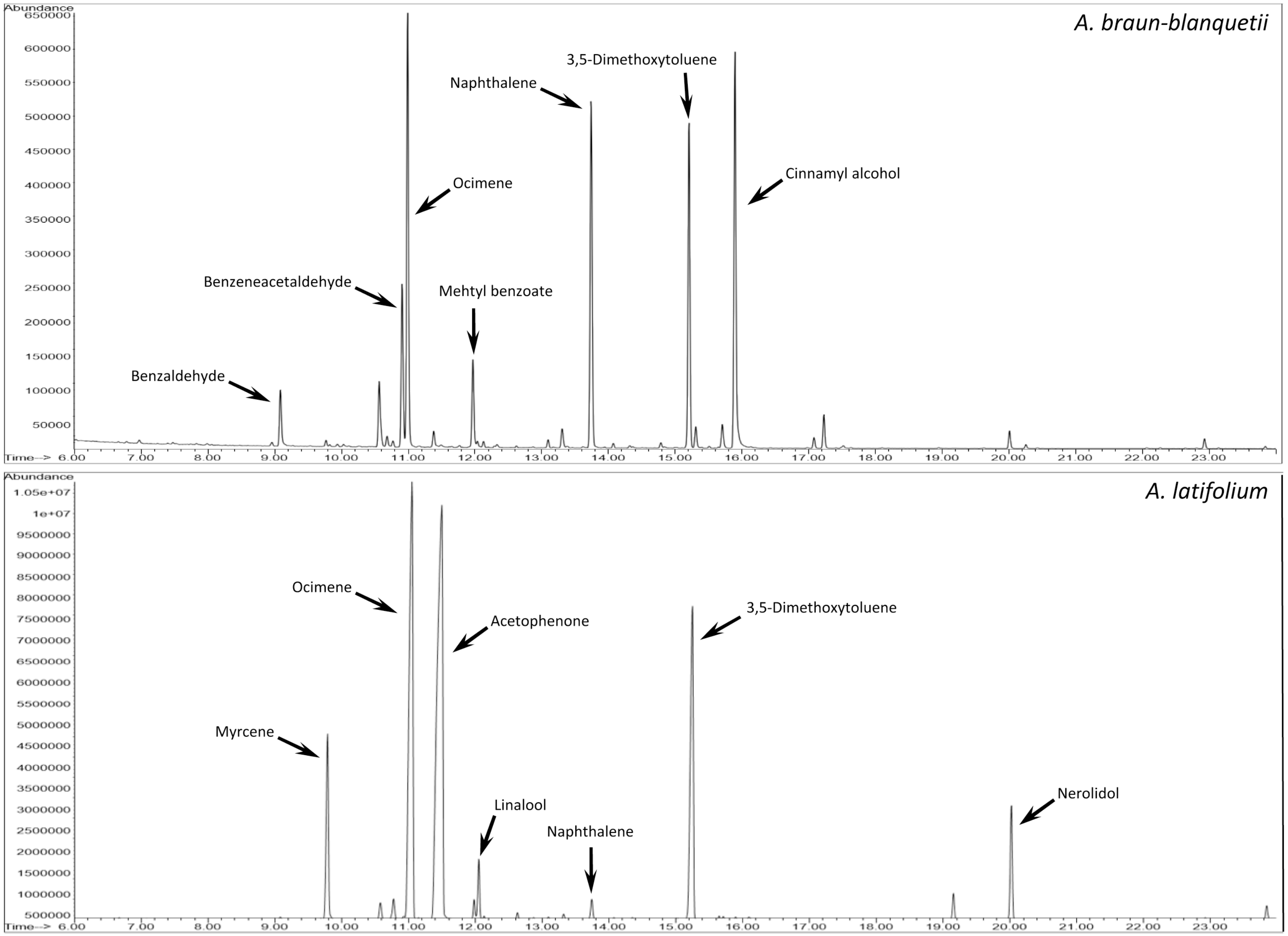

A. braun-blanquetii
Abundance
Benzaldehyde
Benzeneacetaldehyde
Ocimene
Mehtyl benzoate
Naphthalene
3,5-Dimethoxytoluene
Cinnamyl alcohol
Time-->
A. latifolium
Myrcene
Ocimene
Acetophenone
Linalool
Naphthalene
3,5-Dimethoxytoluene
Nerolidol

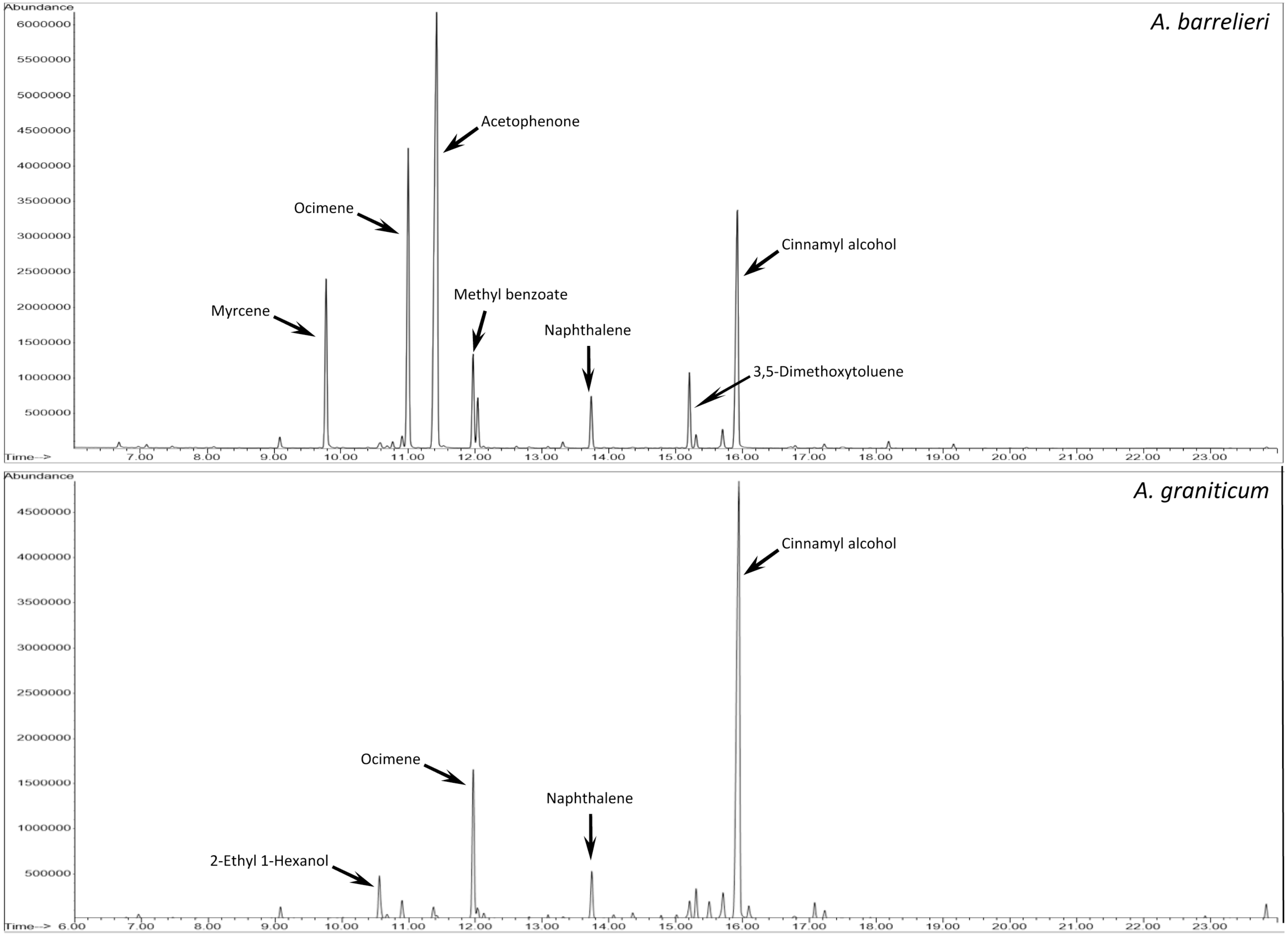
A. barrelieri
Abundance
Time-->
Acetophenone
Ocimene
Cinnamyl alcohol
Myrcene
Methyl benzoate
Naphthalene
3,5-Dimethoxytoluene
A. graniticum
Abundance
Time-->
Cinnamyl alcohol
Ocimene
Naphthalene
2-Ethyl 1-Hexanol
